# Minor Salivary Gland Biopsy in the Differential Diagnosis of Sicca Syndrome: A Monocentric Cohort Analysis

**DOI:** 10.3390/ijms26136463

**Published:** 2025-07-04

**Authors:** Elisa Fiorentini, Pamela Bernardini, Dorilda Zeka, Marco Capassoni, Luca Novelli, Annarita Palomba, Lorenzo Tofani, Laura Cometi, Serena Guiducci

**Affiliations:** 1 Division of Rheumatology, Department of Experimental and Clinical Medicine, University of Florence, AOU Careggi, 50134 Florence, Italy; 2 Department of Services, Laboratory of Pathological Histology and Molecular Diagnostics, University of Florence, AOU Careggi, 50134 Florence, Italy; 3 Department of Statistics, Computer Science, Applications, University of Florence, 50134 Florence, Italy

**Keywords:** Sjögren’s disease, minor salivary gland biopsy, patient-reported outcomes

## Abstract

Sicca syndrome is a common condition that draws the attention of rheumatologists, and is frequently related to Sjögren’s disease (SjD). This study analyzed 164 patients with sicca syndrome (clinically suspected for SjD) who underwent minor salivary gland biopsy (mSGB). Patients completed the Xerostomia Inventory (XI) and Standard Patient Evaluation of Eye Dryness (SPEED) questionnaires to assess Patient-Reported Outcome Measures (PROMs), and biopsies were graded using the Chisholm and Mason system. Patients were classified as seropositive (SSA, SSB, Ro52, Ro60 positive) or seronegative, and also divided into three groups by age. Positive biopsies (60.37%) were more common in older patients (61–80) and associated with confirmed SjD, more severe xerostomia, and stronger lymphocytic infiltrates. Among these, 37.37% were seropositive, showing higher disease activity, hypergammaglobulinemia, and elevated IgG. Seronegative patients had a heavier symptom burden, confirmed by the PROMs, and more fibrosis and fatty replacement in biopsies. Age-stratified analysis showed younger patients (18–40) were more affected by ocular dryness, while older patients had worse xerostomia and more severe histological and ultrasound changes. Younger individuals had higher IgG/IgA, more anemia, and reduced C3. Hydroxychloroquine was used more in younger and seropositive groups; older patients used more topical therapies. These results highlight mSGB’s diagnostic value, especially in seronegative cases, and stress the importance of combining clinical, histological, imaging, and patient-reported outcomes for optimal care.

## 1. Introduction

Sicca syndrome includes symptoms of mucosal dryness, primarily xerostomia (dry mouth) and xerophthalmia (dry eyes), but it can also affect the nose, vagina, and skin. Dryness becomes more common with age and predominantly affects women, with a prevalence in the general population ranging from 5.5% to 46% for xerostomia [1] and 5% to 50% for xerophthalmia [2]. Diagnosing sicca syndrome is complex and requires a detailed medical history to identify the underlying cause and determine the most effective treatment. Possible causes include medication side effects, aging, radiation therapy, viral infections (e.g., hepatitis C, HIV), sarcoidosis, graft-versus-host disease, and systemic autoimmune disorders. Among the latter, Sjögren’s disease (SjD) is the most frequent cause and represents the main focus of this study, conducted in a rheumatological setting.

SjD is a chronic autoimmune disorder that causes inflammation and damage to the lacrimal and salivary glands, resulting in dry eyes and mouth [3]. It can present as either primary SjD or as a secondary condition associated with other autoimmune diseases (e.g., rheumatoid arthritis, systemic lupus erythematosus) [4]. SjD predominantly affects women (in a 9:1 ratio) and is typically diagnosed between the ages of 50 and 60 [5]. Symptoms range from exocrine involvement (e.g., dryness, gland enlargement) to systemic manifestations affecting the skin, joints, lungs, kidneys, nervous system, gastrointestinal tract, and cardiovascular system. SjD is also linked to an increased risk of developing low-grade B-cell lymphomas (e.g., MALT) [3]. Diagnosis of SjD follows the 2016 ACR/EULAR criteria [6], which include chronic dryness (ocular and oral), the presence of anti-SSA/Ro antibodies, hyposalivation, a positive Schirmer test (indicating reduced tear production), and a positive mSGB. The former is a key diagnostic tool for SjD and other infiltrative diseases, such as sarcoidosis, amyloidosis, and lymphomas. For SjD, biopsy sensitivity ranges from 63.5% to 93.7% and specificity from 61.2% to 100% [7]. Various classification systems have been developed to standardize the evaluation of salivary gland biopsies. Chisholm and Mason introduced a five-grade system (0 to 4) based on the extent of lymphocytic infiltration [8]. In 1974, Greenspan and Daniels introduced the concept of focus score (FS), calculated as the number of lymphocytic foci in 4 mm^2^ of glandular tissue [9]. An FS ≥ 1 indicates focal lymphocytic sialadenitis (FLS) and is strongly indicative of SjD. Biopsy also provides valuable prognostic insights. Key histopathological features linked to disease severity and lymphoma risk include massive B-cell infiltration, ectopic germinal centers (GCs)—linked to genetic instability and malignant transformation; lymphoepithelial lesions (LELs)—characterized by lymphocytic infiltration into glandular ducts with epithelial hyperplasia; and altered ratios of IgA+, IgG+, and IgM+ plasma cells—a reduction in IgA+ cells (below 70%) is highly specific for SjD (sensitivity 100%; specificity 95.4%) [10,11,12].

Disease severity in SjD is typically measured using the ESSDAI (EULAR Sjögren’s Syndrome Disease Activity Index) [13]. However, in chronic diseases, it is difficult to fully capture disease activity and treatment response using only biomarkers or clinical parameters. Patients’ subjective symptom perception and expectations significantly impact their quality of life (QoL) [14]. Xerostomia and xerophthalmia can impair daily activities, increase anxiety and depression, and reduce overall well-being. Interestingly, QoL tends to be worse in patients with non-autoimmune sicca syndrome compared to those with SjD, likely due to the uncertainty surrounding diagnosis and treatment options. To better understand the impact of symptoms on patients’ lives, patient-reported outcomes (PROs) are essential tools. They provide insight into the subjective experience of the disease, helping clinicians to tailor treatments and improve patients’ overall well-being [15,16,17,18,19,20,21,22,23,24].

In a rheumatology clinic, patients often report xerostomia and xerophthalmia, and the rheumatologist must perform a proper differential diagnosis. In our department, a diagnostic procedure clinic is active, including mSGB. The aim of this study is to analyze the clinical and histological characteristics of patients undergoing this procedure. The goal is to improve the diagnosis and clinical management of patients.

## 2. Results

This study analyzed a population of 164 patients with a mean age of 59.38 years (±13.16), predominantly female (92.68%) and Caucasian (90.24%). Among the enrolled patients, 54.27% were diagnosed with SjD, with an average disease duration of 0.81 years (±2.07) and a mean ESSDAI score of 1.66 (±3.05). The most frequent symptom was xerostomia, followed by xerophthalmia. Other symptoms related to mucosal dryness, constitutional symptoms, musculoskeletal symptoms, lymphadenopathy, and parotid swelling were less frequently reported (Table 1). All procedures were performed without complications during or after the intervention. No post-biopsy bleeding events requiring sutures or surgical intervention were observed in the study cohort.

### 2.1. Entire Population: Comparison Between Patients with Negative and Positive Biopsies

The entire sample was stratified based on mSGB results: 65 (39.63%) patients had a negative biopsy and 99 (60.37%) had a positive biopsy. Patients with positive biopsies were older (61.29 ± 12.60 vs. 56.48 ± 13.56 years; *p* = 0.0215) and more frequently diagnosed with SjD (79.80% vs. 15.38%; *p* < 0.0001). Smoking habits differed: active smoking was more common in the negative biopsy group (12.31% vs. 3.03%; *p* = 0.0032), while former smokers and non-smokers were more prevalent in the positive biopsy group (Table 2).

ANA positivity was similar in both groups, but the antibody titers were significantly higher in positive biopsies. Anti-centromere antibodies (ACA) and rheumatoid factor (RF) were more common in positive biopsies (19.19% vs. 6.15%; *p* = 0.0213 and 14.29% vs. 1.54%; *p* = 0.0050). Ultrasound of the parotid and submandibular glands showed more moderate and severe changes, according to the OMERACT classification, in the positive biopsy group. Increased levels of beta2-microglobulin, IgM, IgG, and IgA were more common in positive biopsies. Hypergammaglobulinemia was more common in positive biopsies, although the difference was not statistically significant (Table 2).

For symptoms, xerophthalmia and xerostomia were similar between groups, but xerotrachea was more frequent in the positive biopsy group. The Xerostomia Inventory (XI) scores were worse in the positive biopsy group, while the SPEED questionnaire showed no significant overall differences (Table 2).

### 2.2. Positive Biopsies: Comparison Between Seropositive and Seronegative Patients

The second part of the study focused only on patients with positive mSGB, stratified by the presence of SjD-specific antibodies (SSA, SSB, Anti Ro52, Anti Ro60). SjD diagnosis was more frequently confirmed in seropositive patients than in seronegative ones (97.30% vs. 69.35%; *p* = 0.0006). One seropositive patient with a positive biopsy was not classified as having SjD due to the concomitant diagnosis of HCV infection during follow-up. This case was nonetheless retained in the dataset, as the study included all patients referred for mSGB, regardless of the final diagnosis.

Seropositive patients also had higher disease activity (ESSDAI score: 2.26 vs. 0.95; *p* = 0.0079) and slightly longer disease duration (0.72 vs. 0.70 years; *p* = 0.0371). Ultrasound showed more moderate and severe gland changes in seropositive patients. Serologically, seropositive patients had higher levels of ESR, LDH, beta2-microglobulin, hypergammaglobulinemia, IgM, IgG, and IgA (Table 3).

Xerostomia was more common in antibody-negative patients. The Xerostomia Inventory (XI) showed that severe scores were more common in seronegative patients, while mild scores were more frequent in seropositive ones. The SPEED questionnaire showed no significant differences overall, but seronegative patients tended to report more frequent and severe symptoms compared to seropositive ones (Table 3).

Histopathological analysis showed greater lymphocytic infiltration in seropositive patients, predominantly periacinous and periductal, with more CD3+ and CD138+ cells. Seronegative patients had milder interstitial infiltration, with more CD20+ and CD68+ cells. Fibrosis and fatty replacement were more common in antibody-negative patients. IgG4 and high IgG positivity were significantly more frequent in seropositive patients (Table 4).

### 2.3. Entire Population: Comparison Between Age Groups

Finally, we divided the entire sample in three age groups: 18–40, 41–60, and 61–80 years. The older groups had more female and Caucasian patients. SjD diagnosis was more common in older patients but not statistically significant. Weight loss, Raynaud’s phenomenon, asthenia, and inflammatory arthralgia were more frequent in younger patients, while dyspnea was more common in the oldest group. Autoimmune disease history was more frequent in younger patients, while, as expected, older patients had more comorbidities, including osteoporosis, diabetes, hypertension, and history of cancer. Salivary gland ultrasound analysis showed that advanced changes were more common with increasing age. Younger patients more frequently had reduced C3 complement and anemia. In the intermediate group, increased ESR, hypergammaglobulinemia, and elevated IgM and IgG were more common. In older patients, increased beta-2 microglobulin and reduced glomerular filtration rate were more frequently observed (Table 5).

The questionnaire results showed that the average XI and SPEED scores did not differ significantly between age groups, but symptom severity increased with age for the XI questionnaire. Conversely, for the SPEED questionnaire, severe symptoms were more prevalent in younger patients, while moderate symptoms increased with age. Oral and ocular dryness symptoms were more common with age, but the perception of dryness severity declined with age, suggesting possible adaptation or differing perceptions of severity (Table 5).

Histological analysis revealed a trend toward increased biopsy positivity with age, reflecting greater lymphocytic infiltration severity, predominantly periductal, in older groups. The severity of CD3, CD20, CD68, and CD138 infiltrates increased with age. Fibrosis and adipose replacement were more common in older patients, with fibrosis showing distinct patterns by age group: periglandular in younger patients, periacinous in the intermediate group, and interstitial in older patients. IgG4 and IgG positivity also rose with age (Table 6).

### 2.4. Treatments

Hydroxychloroquine (HCQ) was used more frequently in positive biopsy patients and antibody-positive patients, with its use decreasing with age. Methotrexate (MTX) was prescribed predominantly to younger patients, showing a significant age-related difference. Pilocarpine use was low and similar between all the groups. Tear substitutes were more frequently used by younger patients while saliva substitutes were more common in positive biopsy patients and were used more by adults and elderly patients (Table 7).

## 3. Discussion

### 3.1. Role of Age in Histological Changes in Minor Salivary Glands

Patients with positive biopsies were significantly older than those with negative biopsies, and, although not statistically significant, positive biopsy rates increased with age. The relationship between age and FLS remains debated. While some studies found no significant association [25,26,27,28], others reported higher FS in older patients [29,30,31]. A recent Chinese study on autopsy samples noted no significant increase in FLS prevalence until age 60, but found higher FLS rates and FS in those aged 60 and older [32].

### 3.2. Smoking Habits and Histological Changes in Minor Salivary Glands

Patients with negative biopsies showed higher active smoking rates, while those with positive biopsies were more often former smokers or non-smokers. Smoking is linked to higher risk for various diseases, but studies suggest a negative association with SjD development [33,34,35,36,37,38]. Research indicates an inverse relationship between smoking and positive FS. Smoking may influence the immune system by suppressing type 1 interferon, reducing leukocyte chemotaxis, and impairing B-cell differentiation. It is also linked to lower IgA, IgM, and IgG levels in serum and saliva [39,40,41,42].

### 3.3. Correlation Between Histology and Ultrasound

A significant link was found between severe ultrasound abnormalities of major salivary glands and positive mSGB, confirming the established correlation between histology, gland function, and ultrasound findings. Ultrasound is a reliable and widely available tool for early SjD diagnosis and prognosis, useful for monitoring therapy response and lymphoma risk prediction [12,43,44,45,46,47,48]. This correlation held even in older patients, suggesting ultrasound changes reflect histological alterations, although age-related changes might act as confounders. Parotid gland elasticity decreases with age, while submandibular gland elasticity remains stable [49]. Moderate-to-severe ultrasound abnormalities were more common in patients with positive biopsies and SjD-specific autoantibodies (especially anti-Ro52), aligning with other studies. Anti-Ro52 correlates with inflammation severity, suggesting that circulating autoantibodies contribute to local gland damage [44,50,51]. Patients with positive biopsies and autoantibodies showed increased markers of B-cell activation, including higher serum Ig and beta-2 microglobulin, and hypergammaglobulinemia. RF was more frequent in biopsy-positive patients, highlighting its growing prognostic role and its link to increased lymphoma risk due to B-cell hyperactivity [52].

### 3.4. Histological Differences Within Our Cohort

Seropositive patients had higher lymphocytic infiltration, mainly periacinous and periductal, dominated by CD3+ and CD138+. This matches existing findings that lymphocytic infiltrates in SjD can develop into organized structures under the influence of chemokines like CXCL13 and CXCL10. CXCL10, highly expressed in SjD patients, attracts T lymphocytes to glandular tissue, while CXCL13, together with CXCL12, IL-6, and APRIL, supports plasma cell survival and the formation of GCs in infiltrated areas. The periacinous and periductal distribution reflects the key role of the inflamed glandular microenvironment in immune cell organization [10]. IgG4 positivity was frequently observed on immunohistochemistry, particularly in patients with both positive biopsy and SjD-related autoantibodies; however, none fulfilled the diagnostic criteria for IgG4-related disease. This finding may reflect underlying B-cell hyperactivity and immune activation in seropositive individuals with lymphocytic infiltration.

Seronegative patients showed milder and mainly interstitial infiltration, dominated by CD20+ and CD68+, with more fibrosis and fat replacement. This resembles chronic inflammation patterns like nonspecific chronic sialadenitis (NS) and IgG4-related sclerosing sialadenitis (SCS) [53,54,55].

Older patients had increased fibrosis and fat replacement, reflecting age-related glandular deterioration, with reduced acinar volume and increased adipocyte and fibrotic tissue infiltration [56,57]. However, immune and non-immune profibrotic factors likely contribute to this process in SjD patients [58].

### 3.5. mSGB in SjD Diagnosis: The Challenge of Seronegativity

Positive biopsies were more frequently linked to an SjD diagnosis, especially in seropositive patients. In seronegative patients, mSGB was essential for diagnosis. While SjD-specific antibody levels (SSA, SSB, Ro52, Ro60) did not differ significantly between biopsy-positive and -negative patients, ANA titers were higher in biopsy-positive cases.

A subgroup of patients with sicca syndrome, positive biopsy, and ANA positivity but no specific SjD antibodies meet the diagnostic criteria for SjD, and they are called “seronegative SjD” patients, representing 8% to 37% of SjD patients, with a distinct clinical profile [59,60]. Biopsy is crucial for diagnosing seronegative SjD, with almost 30% of seronegative patients testing positive [61]. Advances like AI-based FS analysis could improve diagnostic accuracy [62] and new autoantibodies (targeting DTD2 and RESF1) show predictive potential in seronegative SjD [63]. Some patients may produce autoantibodies only locally, as anti-Ro has been detected in saliva despite negative serum tests [64,65]. Clinically, seronegative SjD patients report more mucosal dryness, arthralgia, and fatigue but show less lymphadenopathy and systemic involvement than seropositive patients, who display higher disease activity and more systemic symptoms [59,66,67]. In our study population, positive biopsy patients reported more intense dryness, especially seronegative ones, while seropositive patients had higher markers of systemic activity (ESR, LDH, beta2-microglobulin, Ig levels, and ESSDAI scores).

### 3.6. Clinical Presentation and Age

Older patients showed a greater frequency and intensity of oral dryness symptoms, while younger patients experienced more severe and prevalent ocular dryness. Interestingly, despite this more severe ocular manifestation in young patients, diagnostic tests like the Schirmer and BUT tests showed increased positivity with age. Older patients also appeared more tolerant to ocular dryness in questionnaires, which contrasts with reports in the literature describing a higher incidence of both types of dryness in older individuals [68,69]. A possible explanation for this discrepancy could be behavioral factors, such as increased screen time and work-related habits among younger patients, which may intensify and worsen symptoms [70].

Younger patients were also more likely to report involuntary weight loss, C3 complement consumption, inflammatory arthralgia, Raynaud’s phenomenon, anemia, family history of autoimmune diseases, and overlap with SLE. It is important to note that the cohort included not only patients with a confirmed SjD diagnosis but also those undergoing salivary gland biopsy due to suspected SjD. Consistent with these findings, early-onset SjD tends to present as a more systemic disease, with greater extra-glandular involvement, higher lymphoma risk, and more frequent hematologic and constitutional symptoms compared to late-onset cases [71].

### 3.7. Therapeutic Approach

HCQ was the most frequently used drug, especially in patients with a positive biopsy and seropositivity, and was more common among younger patients due to the lower risk of maculopathy. Younger patients were also more often treated with MTX because of the higher incidence of inflammatory arthralgia. Pilocarpine was rarely prescribed due to its side effects [72], while saliva substitutes were more common in older patients and tear substitutes in younger ones, reflecting the symptom pattern.

### 3.8. Limitations and Strengths of the Study

A key limitation of our study is the relatively small sample size, which resulted in small comparison groups once the cohort was divided. There were also some missing data, as the collection was based on the diagnostic and therapeutic process of routine clinical practice without a standardized protocol for all patients. Histological analysis was limited to the Chisholm and Mason classification, without evaluation of other elements like FS, ectopic GCs, or LELs, which could have provided a more precise characterization of the lymphocytic infiltrate.

The main study’s strength is the inclusion of the patient perspective, gathered through self-administered questionnaires. This approach allowed for a more direct and detailed evaluation of symptoms, proving more accurate than the VAS scale alone, which is part of the ESSPRI score. This method represents an important contribution to understanding the disease’s impact from the patient’s point of view, enriching the study’s findings.

## 4. Materials and Methods

This study involved patients referred for mSGB at the Rheumatology Department of AOU Careggi in Florence between January 2023 and October 2024. Sicca syndrome was defined as the subjective complaint of dryness (ocular and/or oral) reported by the patient during rheumatological evaluation. This symptom, or the presence of specific autoantibodies associated with SjD (e.g., anti-SSA/Ro or anti-SSB/La), represented the main criterion for referral to salivary gland biopsy. Patients were informed about the biopsy procedure, potential risks, and post-procedure care, and all provided informed consent. Two patients also consented to photographic documentation during the procedure. Anticoagulant or antiplatelet therapy was discontinued in accordance with clinical recommendations prior to biopsy. When discontinuation was not feasible, a local hemostatic sponge was applied to the wound site to promote hemostasis and healing.

Before the biopsy, each patient completed the Xerostomia Inventory (XI) [73] and Standard Patient Evaluation of Eye Dryness (SPEED) [74] questionnaires, as well as numerical Visual Analogue Scales (VAS) to assess dryness in the eyes and mouth, joint pain, and fatigue.

**Figure 1:**
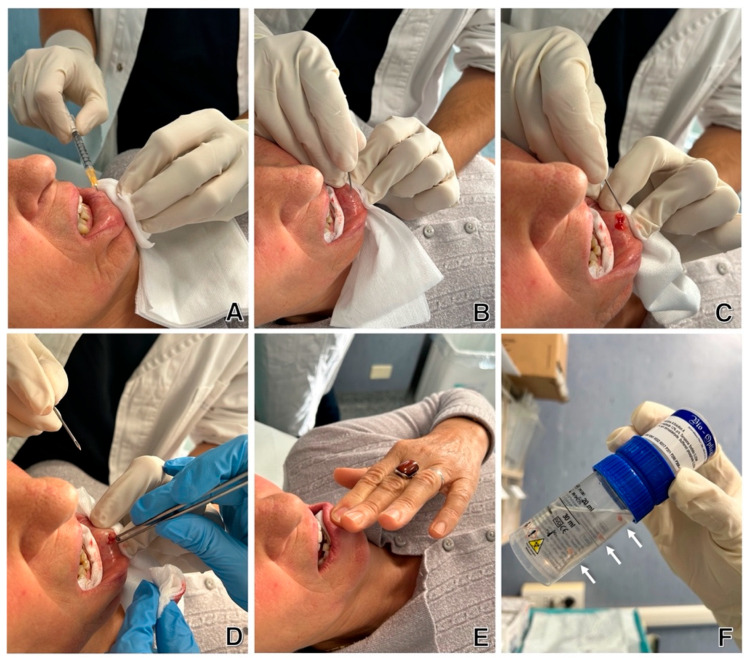
Procedure for performing minor salivary gland biopsy. Original images, acquired with the patient’s consent: (**A**): lidocaine anesthesia; (**B**): incision using an 18G needle tip; (**C**): exposure of the minor salivary glands; (**D**): lifting of the glands with forceps for removal; (**E**): placement of gauze for hemostasis; (**F**): sample in formalin. Arrows indicate the 3 excised glands.

The biopsy was performed by two experienced operators. The patient was positioned supine, and after disinfecting the lower lip mucosa, local anesthesia with 1 mL of lydocaine 2% was administered. A small vertical incision (up to 0.5 cm) was made, allowing the minor salivary glands to emerge. The sample was immediately placed in a container with transport medium, and, if necessary, a second sample was collected (Figure 1).

**Figure 2:**
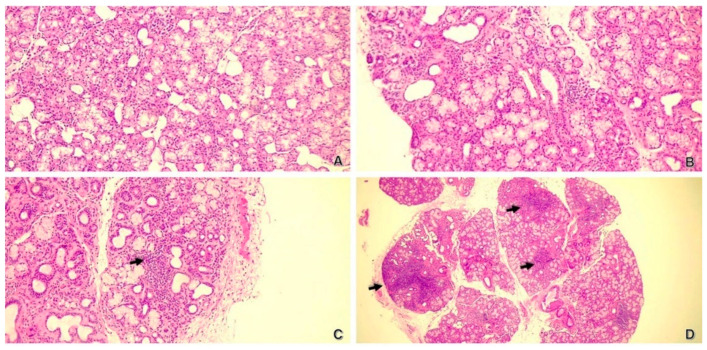
Minor salivary glands in H&E staining, C&M classification. (**A**) (100×): grade 0; (**B**) (100×): grade 1–2; (**C**) (100×): grade 3; (**D**) (25×): grade 4. Black arrows indicate lymphocytic foci.

The specimen was fixed in formalin and processed for histological analysis at the Laboratory of Pathological Histology and Molecular Diagnostics, University of Florence, AOU Careggi. Histological examination included standard hematoxylin–eosin staining; immunohistochemistry for CD20, CD3, CD38, CD68, and IgG4; and additional stains for fibrosis and amyloid. SjD diagnosis was classified using the Chisholm and Mason system, with grades ranging from 0 (no foci) to 4 (severe, multiple foci) (Figure 2).

Data collected included demographic (age, sex, ethnicity), clinical (SjD diagnosis, comorbidities, symptoms), laboratory (autoimmune markers, blood tests), and histological findings (lymphocytic infiltrate, fibrosis, fat replacement). Patient-reported symptoms were assessed with XI, SPEED, and ESSPRI. Treatment details were also documented. Data was collected from electronic medical records within a six-month period before and after the biopsy to ensure consistency with clinical, laboratory, and histological findings.

Diagnosis of SjD was established according to the 2016 ACR/ EULAR classification criteria [6], which require a cumulative score of ≥4 based on clinical symptoms, serological markers, ocular and salivary function tests, and histological findings.

Definitions:

- Diagnosis of SjD: score of ≥4 on the 2016 ACR/EULAR classification criteria [6].
- Positive Biopsy for SjD: Lymphocytic infiltrate of grade 3 or 4 based on C&M classification.
- Negative Biopsy for SjD: Grades 0, 1, or 2 on C&M classification.
- Seropositive Patients: Those with positivity for at least one SjD-specific autoantibody (anti-SSA, anti-SSB, anti-Ro52, anti-Ro60).
- Seronegative Patients: Those without any of these antibodies.

This study developed in three stages: (I) comparing patients with positive and negative biopsy results, (II) analyzing patients with positive biopsies (only grade 3–4 C&M classification) and correlating them with antibody profiles and histological findings, and finally (III) studying age-related differences.

Statistical analysis included *T*-tests, Mann–Whitney tests, Chi-square tests, and Fisher’s exact test, with the significance level set at 5%.

## 5. Conclusions

Our study highlights the importance of mSGB in diagnosing sicca syndrome and SjD, especially in seronegative patients, where it helps reduce underdiagnosis and improve clinical understanding. It confirms a strong link between histological changes and ultrasound findings, reinforcing the value of imaging. Factors like age and smoking were found to influence clinical and histological features. Immunomodulating drugs were more commonly used in younger, seropositive patients, while older patients relied more on topical treatments for dryness. Despite limitations like the small sample size and incomplete histological data, this study’s strength lies in incorporating patient-reported outcomes, enhancing the understanding of disease impact. The results support the routine use of salivary gland biopsy and emphasize integrated diagnostic approaches to improve diagnosis, treatment, and patient quality of life.

## Figures and Tables

**Table 1:** Entire population: demographic and clinical features.

	*n* = 164
**Age (years), medium** (±SD)	59.38 (±13.16)
**Female sex, *n* (%)**	152 (92.68%)
**Ethnicity, *n* (%)** Caucasian Hispanic Asian African American	148 (90.24%) 3 (1.83%) 7 (4.27%) 6 (3.66%)
**Confirmed SjD diagnosis, *n* (%)** Disease duration (years) ESSDAI	89 (54.27%) 0.81 (±2.07) 1.66 (±3.05)
**Xerophthalmia, *n* (%)**	135 (82.32%)
**Positive Schirmer test, *n* (%)**	68 (41.46%)
**Positive Break-Up Time test, *n* (%)**	61 (37.20%)
**Xerostomia, *n* (%)**	144 (87.80%)
**Xerotrachea, *n* (%)**	72 (43.90%)
**Xerovagina, *n* (%)**	50 (30.49%)
**Skin dryness, *n* (%)**	87 (53.05%)
**Itching, *n* (%)**	23 (14.02%)
**Parotid swelling, *n* (%)**	10 (6.10%)
**Fever, *n* (%)**	3 (1.83%)
**Weight loss, *n* (%)**	7 (4.27%)
**Fatigue, *n* (%)**	43 (26.22%)
**Night sweats, *n* (%)**	18 (10.98%)
**Lymphadenopathy, *n* (%)**	61 (37.20%)
**Purpura, *n* (%)**	1 (0.61%)
**Raynaud’s phenomenon, *n* (%)**	47 (28.66%)
**Digital edema, *n* (%)**	9 (5.49%)
**Inflammatory arthralgia, *n* (%)**	18 (10.98%)
**Myalgia, *n* (%)**	37 (22.56%)
**Asthenia, *n* (%)**	31 (19.14%)
**Dyspnea, *n* (%)**	21 (12.80%)

Abbreviations: SD = standard deviation; ESSDAI = EULAR Sjögren’s Syndrome Disease Activity Index.

**Table 2:** Stratification by biopsy outcome: results.

	Negative Biopsies (*n* = 65)	Positive Biopsies (*n* = 99)	*p*-Value
**Age, medium** (±SD)	56.48 (±13.56)	61.29 (±12.60)	**0.0215**
**SjD diagnosis, *n* (%)** Not confirmed Confirmed	55 (84.62%) 10 (15.38%)	20 (20.20%) 79 (79.80%)	**<0.0001**
**Smoking habit, *n* (%)** Never Ongoing Former	50 (76.92%) 8 (12.31%) 7 (10.77%)	81 (81.82%) 3 (3.03%) 15 (15.15%)	**0.0032**
**ANA title, *n* (%)** 1:80 1:160 1:320 1:640 1:1280 1:2560	16 (27.59%) 20 (34.48%) 10 (17.24%) 7 (12.07%) 5 (8.62%) 0 (0%)	5 (6.41%) 19 (24.36%) 17 (21.79%) 15 (19.23%) 17 (21.79%) 5 (6.41%)	**<0.0001**
**SSA** **, *n* (%)**	23 (35.38%)	36 (36.36%)	0.8983
**SSB** **, *n* (%)**	3 (4.62%)	10 (10.1%)	0.2489
**Anti Ro52** **, *n* (%)**	15 (23.08%)	33 (33.33%)	0.1579
**Anti Ro60** **, *n* (%)**	13 (20%)	18 (18.18%)	0.7711
**ACA** **, *n* (%)**	4 (6.15%)	19 (19.19%)	**0.0213**
**RF** **, *n* (%)**	1 (1.54%)	14(14.29%)	**0.0050**
**OMERACT parotid glands, *n* (%)** Normal Minimal change Moderate change Severe change	30 (50.85%) 22 (37.29%) 6 (10.17%) 1 (1.69%)	44 (48.35%) 23 (25.27%) 19 (20.88%) 5 (5.49%)	**0.0007**
**OMERACT submandibular glands, *n* (%)** Normal Minimal change Moderate change Severe change	30 (50.85%) 21 (35.59%) 8 (13.56%) 0 (0%)	37 (40.66%) 29 (31.87%) 21 (23.08%) 4 (4.4%)	**0.0009**
**Beta2-microglobulin increase (>2.5 mg/L)** **, *n* (%)**	9 (13.85%)	22 (22.22%)	**0.0227**
**Hypergammaglobulinemia >19%** **, *n* (%)**	6 (9.23%)	19 (19.19%)	0.0826
**IgM increase (>2.30 g/L)** **, *n* (%)**	1 (1.54%)	4 (4.04%)	**0.0236**
**IgG increase (>16 g/L)** **, *n* (%)**	1 (1.54%)	8 (8.08%)	**0.0049**
**IgA increase (>4.0 g/L)** **, *n* (%)**	1 (1.54%)	2 (2.02%)	**0.0312**
**Xerophthalmia, *n* (%)**	53 (81.54%)	82 (82.83%)	0.8323
**Positive Schirmer test, *n* (%)**	25 (38.46%)	43 (43.43%)	0.3380
**Positive Break-Up Time test, *n* (%)**	23 (35.38%)	38 (38.38%)	0.7221
**Xerostomia, *n* (%)**	55 (84.62%)	89 (89.9%)	0.3118
**Xerotrachea, *n* (%)**	21 (32.31%)	51 (51.52%)	**0.0153**
**XI total score, *n* (%)**	28.55 ± 10.88	30.78 ± 9.92	0.1785
**XI stratified, *n* (%)** Normal (<14) Mild (14–20) Moderate (21–30) Severe (>30)	8 (12.31%) 8 (12.31%) 24 (36.92%) 25 (38.46%)	2 (2.02%) 17 (17.17%) 26 (26.26%) 54 (54.55%)	**<0.0001**
**SPEED total score** **, *n* (%)**	10.82 ± 5.89	10.45 ± 6.00	0.7049
**SPEED stratified, *n* (%)** Mild (0–4) Moderate (5–7) Severe (8–14) Very severe (>15)	10 (15.38%) 8 (12.31%) 29 (44.62%) 18 (27.69%)	16 (16.16%) 18 (18.18%) 42 (42.42%) 23 (23.23%)	0.7471

Abbreviations: SD = standard deviation; SjD = Sjögren’s Disease; ANA = anti-nuclear antibodies; SSA = Anti-Sjögren’s Syndrome A (Ro); SSB = Anti-Sjögren’s Syndrome B (La); ACA = anti-centromere antibodies; RF = rheumatoid factor; OMERACT: Outcome Measures in Rheumatology; IgM = immunoglobulin M; IgG = immunoglobulin G; IgA = immunoglobulin A; XI = Xerostomia Inventory; SPEED = Standardized Patient Evaluation of Eye Dryness. Statistically significant results are in bold.

**Table 3:** Stratification by autoantibodies: results.

	Ab. Negative (*n* = 62)	Ab. Positive (*n* = 37)	*p*-Value
**Age, medium** (±SD)	61.76 (±13.46)	60.51 (±11.14)	0.6369
**SjD diagnosis, *n* (%)** Not confirmed Confirmed	19 (30.65%) 43 (69.35%)	1 (2.7%) 36 (97.3%)	**0.0006**
**Disease duration** **, *n* (%)**	0.70 (±2.37)	0.72 (±1.16)	**0.0371**
**ESSDAI** **, *n* (%)**	0.95 (±2.09)	2.26 (±3.53)	**0.0079**
**ANA positivity** **, *n* (%)**	43 (69.35%)	34 (91.89%)	**0.0116**
**ANA title, *n* (%)** 1:80 1:160 1:320 1:640 1:1280 1:2560	3 (6.98%) 14 (32.56%) 10 (23.26%) 5 (11.63%) 9 (20.93%) 2 (4.65%)	1 (2.7%) 5 (14.29%) 7 (20%) 10 (28.57%) 8 (22.86%) 3 (8.57%)	**<0.0001**
**ACA** **, *n* (%)**	15 (24.19%)	4 (10.81%)	0.1203
**ACPA** **, *n* (%)**	3 (4.84%)	0 (0%)	0.5492
**RF** **, *n* (%)**	6 (9.68%)	8 (22.22%)	0.0871
**OMERACT parotid glands, *n* (%)** Normal Minimal change Moderate change Severe change	29 (51.79%) 15 (26.79%) 9 (16.07%) 3 (5.36%)	15 (42.86%) 8 (22.86%) 10 (28.57%) 2 (5.71%)	**0.0057**
**OMERACT submandibular glands, *n* (%)** Normal Minimal change Moderate change Severe change	22 (39.29%) 23 (41.07%) 10 (17.86%) 1 (1.79%)	15 (42.86%) 6 (17.14%) 11 (31.43%) 3 (8.57%)	**0.0003**
**ESR increase** **, *n* (%)**	10 (16.13%)	13 (35.14%)	**0.0303**
**LDH increase** **, *n* (%)**	1 (1.64%)	5 (13.51%)	**0.0275**
**Beta2-microglobulin increase (>2.5 mg/L)** **, *n* (%)**	10 (16.13%)	12 (32.43%)	**0.0155**
**Hypergammaglobulinemia >19%** **, *n* (%)**	5 (8.06%)	14 (37.84%)	**0.0003**
**IgM increase (>2.30 g/L)** **, *n* (%)**	1 (1.61%)	3 (8.11%)	**0.0225**
**IgG increase (>16 g/L)** **, *n* (%)**	0 (0%)	8 (21.62%)	**<0.0001**
**IgA increase (>4.0 g/L)** **, *n* (%)**	0 (0%)	2 (5.41%)	**0.0148**
**Xerophthalmia, *n* (%)**	53 (85.48%)	29 (78.38%)	0.3644
**Positive Schirmer test, *n* (%)**	29 (46.77%)	14 (37.84%)	0.4888
**Positive Break-Up Time test, *n* (%)**	23 (37.1%)	15 (40.54%)	0.4201
**Xerostomia, *n* (%)**	60 (96.77%)	29 (78.38%)	**0.0052**
**Xerotrachea, *n* (%)**	35 (56.45%)	16 (43.24%)	0.2033
**XI total score, *n* (%)**	32.1 (±9.4)	28.57 (±10.49)	0.0868
**XI stratified, *n* (%)** Normal (<14) Mild (14–20) Moderate (21–30) Severe (>30)	1 (1.61%) 7 (11.29%) 16 (25.81%) 38 (61.29%)	1 (2.7%) 10 (27.03%) 10 (27.03%) 16 (43.24%)	**0.0020**
**SPEED total score** **, *n* (%)**	10.79 (±6.05)	9.89 (±5.96)	0.4741
**SPEED stratified, *n* (%)** Mild (0–4) Moderate (5–7) Severe (8–14) Very severe (>15)	9 (14.52%) 11 (17.74%) 26 (41.94%) 16 (25.81%)	7 (18.92%) 7 (18.92%) 16 (43.24%) 7 (18.92%)	0.8547

Abbreviations: SD = standard deviation; Ab. = antibodies; SjD = Sjögren’s Disease; ESSDAI = EULAR Sjögren’s Syndrome Disease Activity Index; ANA = anti-nuclear antibodies; ACA = anti-centromere antibodies; ACPA = Anti-Cyclic Citrullinated Peptide Antibodies; RF = rheumatoid factor; OMERACT: Outcome Measures in Rheumatology; ESR = Erythrocyte Sedimentation Rate; LDH = Lactate Dehydrogenase; XI = Xerostomia Inventory; SPEED = Standardized Patient Evaluation of Eye Dryness. Statistically significant results are in bold.

**Table 4:** Stratification by autoantibodies: histology.

	Ab. Negative (*n* = 62)	Ab. Positive (*n* = 37)	*p*-value
**Biopsy essential for diagnosis** **, *n* (%)**	43 (69.35%)	32 (86.49%)	**0.0543**
**C&M score, *n* (%)** 3 4	19 (30.65%) 43 (69.35%)	6 (16.22%) 31 (83.78%)	0.1099
**Lymphocytic infiltrates, *n* (%)** Mild Moderate Severe	62 (100%) 23 (37.1%) 28 (45.16%) 11 (17.74%)	37 (100%) 9 (24.32%) 15 (40.54%) 13 (35.14%)	0.1240
**Infiltrates’ localization, *n* (%)** Interstitial Periacinous Periductal Perivascular	53 (85.48%) 31 (50%) 40 (64.52%) 4 (6.45%)	32 (86.49%) 28 (75.68%) 32 (86.49%) 8 (21.62%)	0.8898 **0.0118** **0.0176** **0.0518**
**Infiltrates’type, *n* (%)** **CD3** Absent Mild Moderate Severe Prevalent	2 (3.23%) 26 (41.94%) 1 (1.61%) 0 (0%) 33 (53.23%)	0 (0%) 15 (40.54%) 0 (0%) 1 (2.7%) 21 (56.76%)	**0.0153**
**CD20** Absent Mild Moderate Severe Prevalent	1 (1.61%) 30 (48.39%) 3 (4.84%) 0 (0%) 28 (45.16%)	0 (0%) 17 (45.95%) 4 (10.81%) 0 (0%) 16 (43.24%)	**0.0186**
**CD68** Absent Mild Moderate Severe	8 (12.9%) 48 (77.42%) 6 (9.68%) 0 (0%)	7 (18.92%) 27 (72.97%) 3 (8.11%) 0 (0%)	**0.0460**
**CD138** Absent Mild Moderate Severe	6 (9.68%) 27 (43.55%) 24 (38.71%) 5 (8.06%)	1 (2.7%) 9 (24.32%) 22 (59.46%) 5 (13.51%)	**0.0006**
**Fibrosis, *n* (%)** Mild Moderate Severe	56 (90.32%) 33 (61.11%) 10 (18.52%) 11 (20.37%)	34 (91.89%) 19 (57.58%) 11 (33.33%) 3 (9.09%)	1.0000 **0.0093**
**Fibrosis localization, *n* (%)** Periglandular Periacinous Periductal Interstitial	16 (29.09%) 24 (43.64%) 51 (92.73%) 20 (36.36%)	12 (35.29%) 19 (55.88%) 32 (94.12%) 7 (20.59%)	0.5403 0.2613 1.0000 0.1157
**Fat tissue, *n* (%)** Mild Moderate Severe	47 (75.81%) 33 (70.21%) 9 (19.15%) 5 (10.64%)	27 (72.97%) 22 (81.48%) 2 (7.41%) 3 (11.11%)	0.7536 **0.0341**
**Amiloid** **, *n* (%)**	2 (3.23%)	0 (0%)	0.5271
**IgG4 positivity** **, *n* (%)**	36 (58.06%)	29 (78.38%)	**0.0011**
**IgG positivity, *n* (%)** Mild (<20 HPF) Moderate (20–40 HPF) Severe (>40 HPF)	32 (51.61%) 15 (24.19%) 7 (11.29%)	5 (13.51%) 17 (45.95%) 12 (32.43%)	**<0.0001**

Abbreviations: Ab. = antibodies; C&M score = Chisholm and Mason Score; CD3 = Cluster of Differentiation 3, marker for total T lymphocytes; CD20 = Cluster of Differentiation 20, marker for B lymphocytes; CD68 = Cluster of Differentiation 68, marker for macrophages; CD138 = Cluster of Differentiation 138, marker for plasma cells; IgG = immunoglobulin G; IgG4 = subclass of immunoglobulin G; HPF = High-Power Field, high-magnification microscope field. Statistically significant results are in bold.

**Table 5:** Comparison by age groups: results.

	18–40 (*n* = 18)	41–60 (*n* = 66)	61–80 (*n* = 80)	*p*-Value
**Female** **, *n* (%)**	16 (88.89%)	63 (95.45%)	73 (91.25%)	**0.0425**
**Ethnicity, *n* (%)** Caucasian Hispanic Asian African American	14 (77.78%) 1 (5.56%) 2 (11.11%) 1 (5.56%)	56 (84.85%) 1 (1.52%) 4 (6.06%) 5 (7.58%)	78 (97.5%) 1 (1.25%) 1 (1.25%) 0 (0%)	**<0.0001**
**SjD diagnosis, *n* (%)** Not confirmed Confirmed	13 (72.22%) 5 (27.78%)	29 (43.94%) 37 (56.06%)	33 (41.25%) 47 (58.75%)	**0.0544**
**Weight loss** **, *n* (%)**	2 (11.11%)	1 (1.52%)	4 (5%)	**0.0287**
**Raynaud phenomenon** **, *n* (%)**	10 (55.56%)	18 (27.27%)	19 (23.75%)	**0.0003**
**Inflammatory arthralgia** **, *n* (%)**	4 (22.22%)	7 (10.61%)	7 (8.75%)	**0.0173**
**Myalgia** **, *n* (%)**	4 (22.22%)	17 (25.76%)	1 (20%)	**0.0262**
**Asthenia** **, *n* (%)**	7 (38.89%)	14 (21.54%)	10 (12.66%)	**0.0314**
**Dyspnea** **, *n* (%)**	1 (5.56%)	9 (13.64%)	11 (13.75%)	**0.0167**
**Family history of AIDs** **, *n* (%)**	4 (22.22%)	10 (15.15%)	6 (7.5%)	**0.0080**
**Overlap LES** **, *n* (%)**	1 (5.56%)	4 (6.06%)	1 (1.25%)	**0.0421**
**Osteoporosis** **, *n* (%)**	0 (0%)	6 (9.09%)	15 (18.75%)	**0.0036**
**Hypercholesterolemia** **, *n* (%)**	0 (0%)	16 (24.24%)	28 (35%)	**0.0001**
**Diabetes mellitus** **, *n* (%)**	0 (0%)	1 (1.52%)	6 (7.5%)	**0.0357**
**Hypertension** **, *n* (%)**	0 (0%)	11 (16.67%)	28 (35%)	**<0.0001**
**Cancer history** **, *n* (%)**	0 (0%)	9 (13.64%)	16 (20%)	**0.0045**
**Primary biliary cholangitis** **, *n* (%)**	0 (0%)	0 (0%)	5 (6.25%)	**0.0259**
**OMERACT parotid glands, *n* (%)** Normal Minimal change Moderate change Severe change	11 (68.75%) 3 (18.75%) 2 (12.5%) 0 (0%)	23 (38.33%) 23 (38.33%) 12 (20%) 2 (3.33%)	40 (54.05%) 19 (25.68%) 11 (14.86%) 4 (5.41%)	**<0.0001**
**OMERACT submandibular glands, *n* (%)** Normal Minimal change Moderate change Severe change	12 (75%) 3 (18.75%) 1 (6.25%) 0 (0%)	26 (43.33%) 20 (33.33%) 12 (20) 2 (3.33%)	29 (39.19%) 27 (36.49%) 16 (21.62%) 2 (2.7%)	**<0.0001**
**ESR increase** **, *n* (%)**	1 (5.56%)	16 (24.24%)	18 (22.5%)	**0.0086**
**C3 reduction, *n* (%)**	6 (33.33%)	15 (22.73%)	8 (10%)	**0.0244**
**Beta2-microglobulin increase (>2.5 mg/L), *n* (%)**	1(5.56%)	11(16.67%)	19(23.75%)	**0.0009**
**Hypergammaglobulinemia >19%, *n* (%)**	2 (11.11%)	16 (24.24%)	7(8.75%)	**0.0019**
**IgM increase (>2.30 g/L), *n* (%)**	0 (0%)	4 (6.06%)	1 (1.25%)	**0.0016**
**IgG increase (>16 g/L), *n* (%)**	1 (5.56%)	6 (9.09%)	2 (2.5%)	**0.0004**
**IgA increase (>4.0 g/L), *n* (%)**	1 (5.56%)	1 (1.52%)	1 (1.25%)	**0.0008**
**Anemia, *n* (%)**	2 (11.11%)	3 (3.03%)	1 (1.25%)	**0.0282**
**CrCl reduction, *n* (%)**	0 (0%)	1 (1.52%)	12 (15%)	**<0.0001**
**Xerophthalmia, *n* (%)**	17 (94.44%)	55 (83.33%)	63 (78.75%)	**0.0142**
**Positive Schirmer test, *n* (%)**	5 (27.78%)	26 (39.39%)	37 (46.25%)	**0.0005**
**Positive BUT test, *n* (%)**	5 (27.78%)	22 (33.33%)	34 (42.5%)	**0.0002**
**Xerostomia, *n* (%)**	14 (77.78%)	55 (83.33%)	75 (93.75%)	**0.0032**
**XI total score, *n* (%)**	30.83 (±12.37)	31.2 (±10.04)	38.06 (±9.98)	0.1739
**XI stratified, *n* (%)** Normal (<14) Mild (14–20) Moderate (21–30) Severe (>30)	1 (5.56%) 4 (22.22%) 4 (22.22%) 9 (50%)	5 (7.58%) 10 (15.15%) 25 (37.88%) 26 (39.39%)	4 (5%) 11 (13.75%) 21 (26.25%) 44 (55%)	**<0.0001**
**SPEED total score, *n* (%)**	12.83 (±6.11)	10.83 (±5.25)	9.9 (±5.88)	0.1532
**SPEED stratified, *n* (%)** Mild (0–4) Moderate (5–7) Severe (8–14) Very severe (>15)	1 (5.56%) 2 (11.11%) 8 (44.44%) 7 (38.89%)	11 (16.67%) 8 (12.12%) 29 (43.94%) 18 (27.27%)	14 (17.5%) 16 (20%) 34 (42.5%) 16 (20%)	**<0.0001**

Abbreviations: SjD = Sjögren’s Disease; OMERACT: Outcome Measures in Rheumatology; ESR = Erythrocyte Sedimentation Rate; XI = Xerostomia Inventory; SPEED = Standardized Patient Evaluation of Eye Dryness. Statistically significant results are in bold.

**Table 6:** Comparison by age groups: histology.

	18–40 (*n* = 18)	41–60 (*n* = 66)	61–80 (*n* = 80)	*p*-Value
**Positive biopsy, *n* (%)**	7 (38.89%)	39 (59.09%)	53 (66.25%)	0.0967
**Biopsy essential for diagnosis, *n* (%)**	4 (50%)	31 (70.45%)	40 (67.8%)	**0.0266**
**C&M score, *n* (%)** **0** **1** **2** 3 4	0 (0%) 7 (38.89%) 4 (22.22%) 3 (16.67%) 4 (22.22%)	1 (1.52%) 21 (31.82%) 5 (7.58%) 8 (12.12%) 31 (46.97%)	0 0(0%) 20 (25%) 7 (8.75%) 14 (17.5%) 39 (48.75%)	**<0.0001**
**Lymphocytic infiltrates, *n* (%)** Mild Moderate Severe	18 (100%) 14 (77.78%) 2 (11.11%) 2 (11.11%)	65 (98.48%) 38 (58.46%) 12 (18.46%) 15 (23.08%)	80 (100%) 44 (55%) 29 (36.25%) 7 (8.75%)	0.4024 **<0.0001**
**Infiltrates’ localization, *n* (%)** Interstitial Periacinous Periductal Perivascular	16 (88.89%) 6 (33.33%) 4 (22.22%) 0 (0%)	58 (89.23%) 25 (38.46%) 28 (43.08%) 6 (9.23%)	71 (88.75%) 29 (36.25%) 46 (57.5%) 6 (7.5%)	0.0635 0.9137 **0.0004** 0.0512
**Infiltrates’ type, *n* (%)** **CD3** Absent Mild Moderate Severe Prevalent	8 (44.44%) 4 (22.22%) 0 (0%) 0 (0%) 6 (33.33%)	25 (38.46%) 14 (21.54%) 0 (0%) 1 (1.54%) 25 (38.46%)	21 (26.25%) 30 (37.5%) 1 (1.25%) 0 (0%) 28 (35%)	**<0.0001**
**CD20** Absent Mild Moderate Severe Prevalent	12 (66.67%) 3 (16.67%) 2 (11.11%) 0 (0%) 1 (5.56%)	26 (40%) 21 (32.31%) 4 (6.15%) 0 (0%) 14 (21.54%)	23 (28.75%) 27 (33.75%) 1 (1.25%) 0 (0%) 29 (36.25%)	**<0.0001**
**CD68** Absent Mild Moderate Severe	13 (72.22%) 5 (27.78%) 0 (0%) 0 (0%)	30 (46.15%) 33 (50.77%) 2 (3.08%) 0 (0%)	35 (43.75%) 38 (47.5%) 7 (8.75%) 0 (0%)	**0.0002**
**CD138** Absent Mild Moderate Severe	12 (66.67%) 5 (27.78%) 0 (0%) 1 (5.56%)	30 (46.15%) 9 (13.85%) 20 (30.77%) 6 (9.23%)	28 (35%) 23 (28.75%) 26 (32.5%) 3 (3.75%)	**<0.0001**
**Fibrosis, *n* (%)** Mild Moderate Severe	6 (33.33%) 5 (100%) 0 (0%) 0 (0%)	37 (56.06%) 22 (62.86%) 10 (28.57%) 3 (8.57%)	56 (70%) 32 (57.14%) 13 (23.21%) 11 (19.64%)	**0.0105**
**Fibrosis localization, *n* (%)** Periglandular Periacinous Periductal Interstitial	3 (50%) 3 (50%) 5 (83.33%) 1 (16.67%)	11 (29.73%) 20 (54.05%) 34 (91.89%) 9 (24.32%)	20 (36.36%) 27 (49.09%) 50 (90.91%) 18 (32.73%)	**0.0343** **0.0487** 0.1031 **0.0417**
**Fat tissue, *n* (%)** Mild Moderate Severe	3 (16.67%) 3 (100%) 0 (0%) 0 (0%)	30 (45.45%) 22 (75.86%) 5 (17.24%) 2 (6.9%)	47 (58.75%) 34 (70.83%) 7 (14.58%) 7 (14.58%)	**0.0001** **0.0183**
**Amiloid, *n* (%)**	0 (0%)	1 (1.52%)	1 (1.25%)	0.3950
**IgG4 positivity, *n* (%)**	2 (11.11%)	28 (42.42%)	35 (43.75%)	**0.0001**
**IgG positivity, *n* (%)** Mild (<20 HPF) Moderate (20–40 HPF) Severe (>40 HPF)	2 (11.11%) 3 (16.67%) 0 (0%)	12 (18.18%) 13 (19.7%) 10 (15.15%)	24 (30%) 16 (20%) 9 (11.25%)	**<0.0001**

Abbreviations: C&M score = Chisholm and Mason Score; CD3 = Cluster of Differentiation 3, marker for total T lymphocytes; CD20 = Cluster of Differentiation 20, marker for B lymphocytes; CD68 = Cluster of Differentiation 68, marker for macrophages; CD138 = Cluster of Differentiation 138, marker for plasma cells; IgG = immunoglobulin G; IgG4 = subclass of immunoglobulin G; HPF = High-Power Field, high-magnification microscope field. Statistically significant results are in bold

**Table 7:** Treatments.

	Neg. (65)	Pos. (99)	*p*-Value	Ab − (62)	Ab + (37)	*p*-Value	18–40 (18)	41–60 (66)	61–80 (80)	*p*-Value
**HCQ, *n* (%)**	17 (26.15%)	56 (56.57%)	**0.0003**	27 (43.55%)	29 (78.38%)	**<0.0001**	10 (55.56%)	32 (48.48%)	31 (38.75%)	**0.0004**
**MTX, *n* (%)**	0 (0%)	2 (2.02%)	0.0769	1 (1.61%)	1 (2.7%)	0.2113	1 (5.56%)	0 (0%)	1 (1.25%)	**0.0071**
**LEF, *n* (%)**	1 (1.54%)	1 (1.01%)	1.0000	0 (0%)	1 (2.7%)	0.3737	0 (0%)	1 (1.52%)	1 (1.25%)	0.3950
**AZA, *n* (%)**	1 (1.54%)	0 (0%)	0.3963	0 (0%)	0 (0%)	0.3737	0 (0%)	1 (1.52%)	0 (0%)	0.4024
**MMF, *n* (%)**	0 (0%)	2 (2.02%)	0.5186	2 (3.23%)	0 (0%)	0.5271	0 (0%)	0 (0%)	2 (2.5%)	0.2364
**RTX, *n* (%)**	0 (0%)	1 (1.01%)	1.0000	0 (0%)	1 (2.7%)	0.3737	0 (0%)	1 (1.52%)	0 (0%)	0.4024
**Pilo., *n* (%)**	2 (3.08%)	2 (2.02%)	0.6491	2 (3.23%)	0 (0%)	0.5271	0 (0%)	1 (1.52%)	3 (3.75%)	0.1867
**CCS, *n* (%)**	0 (0%)	3 (3.03%)	0.1053	2 (3.23%)	1 (2.7%)	0.2788	0 (0%)	2 (3.03%)	1 (1.25%)	0.0569
**Tears sub., *n* (%)**	53 (81.54%)	82 (82.83%)	0.8323	52 (83.87%)	27 (72.97%)	0.1914	16 (88.89%)	54 (81.82%)	62 (77.5%)	**0.0239**
**Saliva su, *n* (%)b.**	10 (15.38%)	28 (28.28%)	**0.0555**	17 (27.42%)	11 (29.73%)	0.8050	2 (11.11%)	16 (24.24%)	20 (25%)	**0.0175**

Abbreviations: Neg.: negative; Pos.: positive; Ab.: antibodies; HCQ = Hydroxychloroquine; MTX = Methotrexate; LEF = Leflunomide; AZA = Azatioprine; MMF = Micofenolate Mofetile; RTX = Rituximab; Pilo: Pilocarpine; CCS: corticosteroids; Sub.: substitutes. Statistically significant results are in bold

## Data Availability

The raw data supporting the conclusions of this article will be made available by the authors on request.
